# Intelligent Laser Micro/Nano Processing: Research and Advances

**DOI:** 10.3390/nano15191462

**Published:** 2025-09-23

**Authors:** Yu-Xin Liu, Wei Gong, Fan-Gao Bu, Xin-Jing Zhao, Song Li, Wei-Wei Xu, Ai-Wu Li, Guo-Hong Liu, Tao An, Bing-Rong Gao

**Affiliations:** 1 State Key Laboratory of Integrated Optoelectronics, College of Electronic Science and Engineering, Jilin University, Changchun 130012, China; yxliu1922@mails.jlu.edu.cn (Y.-X.L.); gongwei24@mails.jlu.edu.cn (W.G.); bufg24@mails.jlu.edu.cn (F.-G.B.); zhaoxj22@mails.jlu.edu.cn (X.-J.Z.); liaw@jlu.edu.cn (A.-W.L.); 2 College of Science, Laboratory of Materials Design and Quantum Simulation, Changchun University, 6543 Weixing Road, Changchun 130022, China; 240501274@mails.ccu.edu.cn (S.L.); ant@ccu.edu.cn (T.A.); 3 School of Electrical and Information Engineering, Jilin Engineering Normal University, Changchun 130052, China; 4 College of Communication Engineering, Jilin University, Changchun 130022, China; graceliu@jlu.edu.cn

**Keywords:** laser micro/nano processing, machine learning, predictive modeling, in situ detection

## Abstract

Artificial intelligence (AI), particularly machine learning (ML), is equipping laser micro/nano processing with significant intelligent capabilities, demonstrating exceptional performance in areas such as manufacturing process modeling, process parameter optimization, and real-time anomaly detection. This transformative potential is driving the development of next-generation laser micro/nano processing technologies. The key challenges confronting traditional laser manufacturing stem from the complexity of laser–matter interactions, resulting in difficult-to-control processing outcomes and the accumulation of micro/nano defects across multi-step processes, ultimately triggering catastrophic process failures. This review provides an in-depth exploration of how machine learning effectively addresses these challenges through the integration of data-driven modeling with physics-driven modeling, coupled with intelligent in situ monitoring and adaptive control techniques. Systematically, we summarize current representative breakthroughs and frontier advances at the intersection of machine learning and laser micro/nano processing research. Furthermore, we outline potential future research directions and promising application prospects within this interdisciplinary field.

## 1. Introduction

Laser micro/nano processing has become an indispensable technology in the field of high-precision manufacturing for its unique advantages, including no material selectivity, three-dimensional processing, and rapid removal [1,2,3,4,5]. For example, the technique can create coronary stents with nanometer surface textures to enhance biocompatibility far beyond electrochemical polishing capabilities, which has revolutionized medical device production [1,2]. In aerospace turbine blades, the technique has been used to fabricate ten-micrometer-wide cooling channels that work at several hundred centigrade [3]. In microelectronics, laser processing (especially femtosecond lasers) can create sub-wavelength optical interconnects that outperform traditional lithography in three-dimensional integration [4,5].

**Figure 1:**
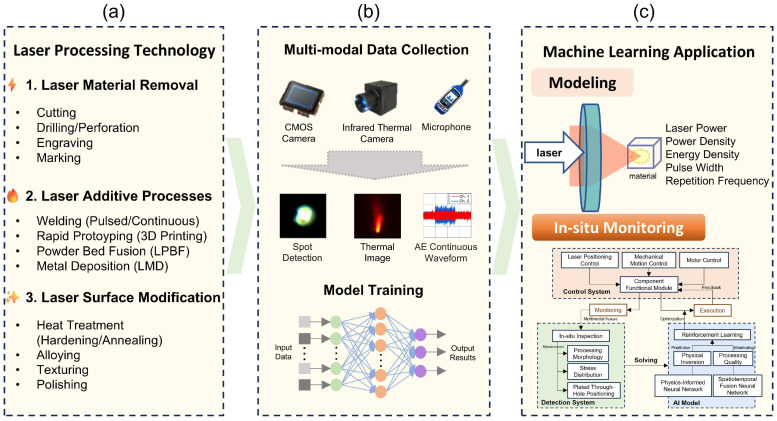
(**a**) Application fields of laser processing. (**b**) Multimodal Data Collection and Model Training Process. Adapted with permission from Ref. [23]. Copyright 2025, Laser Institute of America. Ref. [24]. Copyright 2023, Elsevier; Ref. [25]. Copyright 2021, Elsevier. (**c**) Application of machine learning in laser processing.

As shown in Figure 1a, laser processing technology encompasses three principal methodologies, namely additive manufacturing, e.g., laser powder bed fusion for metallic components [6,7,8], subtractive techniques [9,10,11,12,13], e.g., precision cutting of composites, and surface modification [14,15], e.g., laser texturing of biomaterials. Despite their diverse applications, the techniques all share a fundamental process to transfer laser energy to a material. During this process, the absorption coefficients and thermal conductivity define the photon–matter interaction dynamics (Figure 1b) [16]. In other words, the laser parameters (wavelength, pulse energy, etc.) and material parameters (properties) collectively determine this process. Computational models subsequently predict processing outcomes based on these parameters through multi-physics simulations (Figure 1c). During material transformation, dynamic phenomena including plasma formation and melt pool oscillations occur within microsecond timescales while concurrent in situ monitoring systems capture these events using high-speed imaging [17,18] and acoustic sensors [19,20]. Real-time feedback mechanisms then analyze sensor data streams to identify deviations like thermal anomalies or geometric inaccuracies and instantaneously adjust laser power or focal position to compensate for disturbances such as material inhomogeneity, thereby preventing defect propagation [21,22].

**Figure 2:**
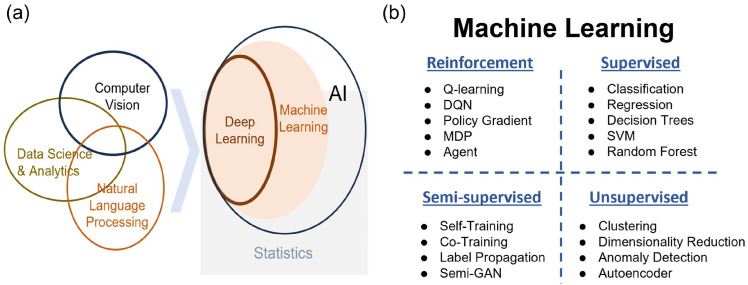
(**a**) Concept map of machine learning domains; (**b**) taxonomy of machine learning.

Significant challenges persist due to the intricate physics in which the nonlinear energy absorption stochastic plasma effects [26] and quantum-level phenomena create unpredictable outcomes. Traditional modeling approaches struggle to accurately simulate these complex multi-scale processes, leading to quality control difficulties. Furthermore, cascading failures emerge when micron-scale defects such as subsurface porosity in additively manufactured parts induce catastrophic failures during subsequent thermal processing stages [27]. These dual limitations necessitate artificial intelligence solutions. In previous research, machine learning has been applied to a wide range of applications in optics, such as holographic displays [28], fluorescence imaging [29], ultrafast laser system design [30], optimization of lasers [31,32], confocal 3D imaging speed-ups [33], and hypersurface design [34]. As shown in Figure 2, four machine learning paradigms demonstrate distinct capabilities [35]. Supervised learning establishes correlations between input parameters and quality metrics using labeled experimental data, enabling predictive modeling for process optimization [36]. Semi-supervised learning combines limited annotations with abundant unlabeled sensor data to reduce the costly annotation requirements for anomaly detection systems [37,38]. Unsupervised learning autonomously identifies hidden process patterns from raw sensor signals, facilitating real-time monitoring without predefined defect signatures [39,40]. Reinforcement learning implements adaptive control strategies that dynamically adjust processing parameters based on environmental feedback to suppress error propagation across manufacturing stages [41,42,43,44].

To better illustrate how machine learning can empower the various processes of laser micro/nano processing, we divide laser micro/nano processing into three processes: modeling [45,46,47,48,49,50,51,52], in situ monitoring [17,18,53,54,55,56], and feedback control [22,57]. Different machine learning methods are involved in different laser micro/nano processing processes. For example, convolutional neural networks (CNNs) are often used for high-precision real-time image analysis [53], and reinforcement learning (RL) is often used for feedback control processes [22]. Table 1 summarizes the machine learning algorithms commonly used in laser micro/nano processing research for each laser processing process and their characteristics, for the convenience of readers [17,18,22,45,46,47,48,49,50,51,52,53,54,55,56,57].

This review systematically synthesizes machine learning’s transformative role across laser micro/nano processing. It examines how data-driven and physics-driven modeling converge to overcome complexities in predicting multi-physics phenomena like melt pool hydrodynamics and vapor plume interactions. Furthermore, we discuss intelligent monitoring frameworks where optical imaging [58], acoustic emission analysis [19], laser interferometry [56], thermal sensing [59], and multi-modal fusion collectively [60] achieve unprecedented resolution in defect detection. Crucially, these advancements enable closed-loop control systems [61,62,63,64] that translate real-time sensor insights into adaptive laser parameter adjustments, resolving challenges in process stability and defect accumulation [65,66,67,68,69]. By integrating these interconnected domains, this article summarizes and looks ahead to future development paradigms in the field of intelligent laser micro/nano processing.

## 2. Machine Learning-Assisted Modeling Workflow

Laser processing technology occupies a pivotal position in advanced manufacturing due to its high precision, efficiency, non-contact nature, and exceptional process flexibility. However, the physical process of laser–material interaction involves complex nonlinear thermodynamic effects, hydrodynamic behaviors, and phase transformations, whose intrinsic mechanisms are highly intricate and influenced by the coupled effects of numerous process parameters (e.g., laser power, scan speed, spot size, defocus distance, and shielding gas) and environmental factors [70]. Traditional physics-based analytical models or numerical simulations, while possessing clear physical significance, face significant challenges in accurately characterizing transient, multi-scale, multi-physics phenomena during actual processing. These include prohibitive computational costs, oversimplified model assumptions, and difficulties in real-time implementation. In recent years, the rapid advancement of machine learning technology has provided new opportunities to overcome these bottlenecks, opening innovative pathways for constructing efficient and high-fidelity laser processing models. The core advantage of machine learning-assisted modeling lies in its powerful capability to learn complex nonlinear relationships from data, effectively capturing mapping correlations between process parameters, in-process states, and final quality metrics, such as melt pool morphology, heat-affected zones, residual stress, geometric accuracy, surface roughness, and defect formation [47], thereby enabling prediction, optimization, and control of processing outcomes.

Based on the utilization level of physical prior knowledge, machine learning-assisted laser processing modeling can be categorized into two primary paradigms: data-driven modeling and physics-driven modeling. The data-driven paradigm primarily relies on extensive process data acquired from experiments or sensor monitoring (e.g., high-speed camera-recorded melt pool images, infrared thermography-captured temperature fields, acoustic emission signals, spectral data, and online monitoring streams) [71]. It employs supervised, unsupervised, or reinforcement learning algorithms to directly mine statistical correlations between inputs (process parameters/signals) and outputs (quality metrics), constructing “black-box” or “grey-box” predictive models. Its essence lies in revealing latent patterns through data itself, with minimal explicit reliance on physical mechanisms. In contrast, the physics-informed paradigm embeds known physical laws (e.g., energy/mass/momentum conservation, heat conduction equations, hydrodynamic equations) as soft constraints (e.g., loss function terms) or hard constraints (e.g., network architecture designs) into the machine learning model construction process [72]. This approach not only leverages observational data but also fully incorporates prior knowledge describing fundamental physical processes, guiding the model’s learning toward greater physical consistency. Consequently, it enhances model generalizability and interpretability and reduces dependency on massive high-quality datasets. These two paradigms, each with distinct emphases, collectively form a versatile toolbox for machine learning-empowered comprehension and prediction of laser processing, laying a solid foundation for in-depth analysis of complex mechanisms and intelligent process optimization with real-time monitoring.

### 2.1. Data-Driven Modeling

Data-driven modeling leverages machine learning (ML) to directly extract intricate nonlinear relationships between laser processing parameters (e.g., power, scan speed) and outcomes (e.g., melt pool dynamics, surface quality) from experimental datasets, bypassing explicit physical equations—particularly valuable in scenarios where abundant data compensate for mechanistic opacity. By training on large-scale datasets, models like ANNs [46] and GANs [73] achieve high-precision predictions. We critically examine how these methods distill actionable insights from heterogeneous datasets, enabling rapid exploration of process–microstructure–property relationships beyond traditional physics-based constraints, while analyzing their advantages and limitations.

**Figure 3:**
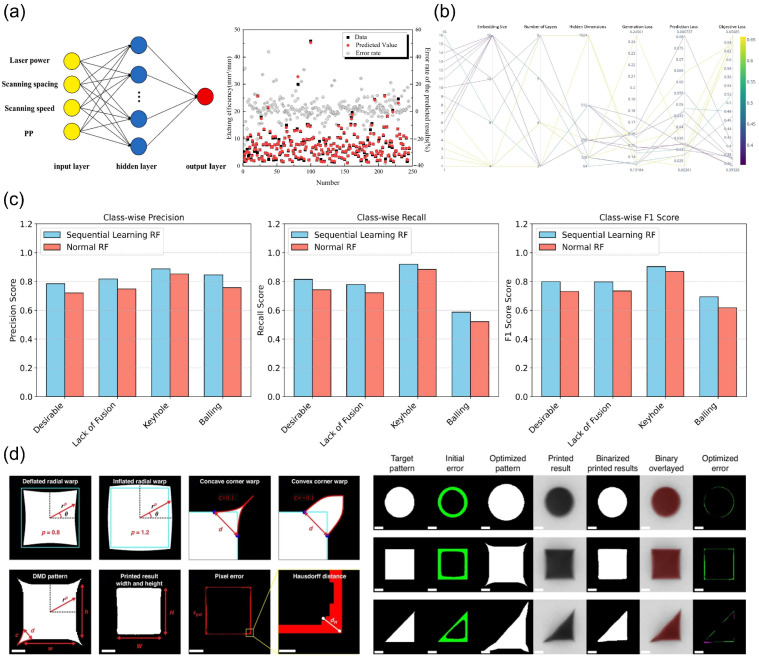
(**a**) Establishment and training results of ANN model [46]; (**b**) model optimization procedure [47]; (**c**) performance comparison between SL-RF+ and conventional RF models; Reprinted with permission from Ref. [74]. Copyright 2025, Elsevier; (**d**) experimental process of target shape adjustment via GPR-based active learning framework [45].

Zhang et al. proposed a method integrating femtosecond lasers and machine learning for laser drilling, establishing an artificial neural network (ANN) model to successfully predict drilling quality and efficiency [46]. For quality prediction, Zhang et al. developed an ANN model comprising an input layer, a hidden layer (32 neurons), and an output layer. Input parameters included laser power, scan spacing, scan speed, and pulse number (PP), with outputs classified as “good” or “poor” drilling quality. The model achieved 98% accuracy on the test dataset. Additionally, Zhang et al. constructed another ANN model for drilling efficiency prediction, demonstrating exceptional performance with a correlation coefficient (R^2^) of 0.99681. Figure 3a illustrates the training results of the ANN model for laser processing efficiency, showing high consistency between predicted and experimental results. Ferreira et al. introduced the Gen-JEMA method, which learns semantic representations by integrating multi-modal data (e.g., images and process parameters), enhancing feature extraction and prediction interpretability [47]. In directed energy deposition (DED), Gen-JEMA significantly improved performance, particularly in melt pool geometry prediction and external melt pool representation generation. Through Bayesian optimization, the autoencoder (AE) architecture achieved a 38% improvement in melt pool geometry prediction and an 88% enhancement in data generation, while the variational autoencoder (VAE) performed slightly inferior. Figure 3b depicts the impact of different parameter combinations on evaluation metrics during neural architecture search (NAS), indicating reduced final objective loss with increasing trial iterations, thereby optimizing model performance.

Raihan et al. proposed the SL-RF+ framework, combining random forests, learning classifier systems (LCSs), and Sobol sequence sampling to enhance efficiency and accuracy in melt pool defect classification for laser powder bed fusion (L-PBF) [74]. SL-RF+ optimizes decision boundaries by iteratively selecting informative samples, excelling in data-scarce environments. As shown in Figure 3c, SL-RF+ outperforms traditional RF models in defect classification precision, recall, and F1 score, particularly for “lack of fusion” and “ideal melt pool” categories, significantly reducing false positives. Recall rates for “lack of fusion” and “balling” defects were equally outstanding, effectively identifying true defect instances. SL-RF+ improves classification performance while reducing labeled samples, enabling accurate prediction and optimization of laser processing under limited data conditions. Johnson et al. developed a Gaussian process regression (GPR)-based active learning framework to optimize geometric accuracy in projection multiphoton 3D printing [45]. The framework collects experimental data via Bayesian optimization (BO) to train a multi-output GPR model for predicting and optimizing 2D geometries. Figure 3d demonstrates the experimental process adjusting target shapes using five input parameters (width, height, radial warping, etc.). After four BO iterations, geometric errors significantly decreased, reaching measurement precision. This highlights ML’s potential in laser processing modeling for enhancing 3D printing accuracy through parameter optimization.

Vagheesan et al. employed a hybrid ANN-GA/PSO framework to optimize CO_2_ laser cutting parameters (power, speed, gas pressure) for Al6061 alloy [75]. By integrating artificial neural networks with evolutionary algorithms, they addressed multi-objective optimization challenges (minimizing kerf taper/surface roughness while ensuring > 4 μm depth), reducing experimental trials by 30%. The ANN acts as a surrogate model predicting kerf geometry, while GA/PSO navigates high-dimensional parameter spaces. However, the approach neglects thermal accumulation effects in multi-pulse processing and cannot resolve spatial heterogeneity in melt pool dynamics, limiting applicability to complex 3D structures. McDonnell et al. developed progressive growing GANs (PGGANs) for spatiotemporal beam shaping in femtosecond nickel machining [73]. By training on interferometric depth profiles, the generator synthesizes 3D surface topographies from DMD pulse sequences with 1.01 nm MAE. This architecture incorporates noise inputs to handle non-deterministic material responses, enabling predictive visualization of multi-pulse interactions. Limitations include homogeneous material assumptions (electroless nickel) and black-box obscuration of diffraction compensation mechanisms, restricting applicability to anisotropic alloys. In the same year, McDonnell et al. also pioneered conditional GANs with SPADE ResBlocks to solve the inverse problem: generating DMD patterns from target depth profiles [76]. This framework addresses the ill-posed inverse mapping challenge through pulse-weighting constraints and cycle-consistent losses, enabling controllable multi-pulse ablation sequences. While achieving 14.9% depth error reduction versus direct conversion methods, unresolved thermal gradient effects in ultrafast interactions limit accuracy. The method demonstrates particular strength in compensating for diffraction artifacts through edge-enhancement patterns. Zhang et al. (2021) developed a data-driven predictive model utilizing machine learning algorithms, extreme gradient boosting (XGBoost) and long short-term memory (LSTM), to forecast the melt pool temperature during the directed energy deposition (DED) process [77]. Experimental validations demonstrated high accuracy in predicting melt pool temperatures, with LSTM exhibiting lower prediction errors in cases of minimal temperature fluctuations, while XGBoost showed superior computational efficiency. This research provides an effective approach to enhancing the predictive accuracy of microstructure, porosity, and mechanical properties of DED-fabricated metal components.

Carbon-fiber-reinforced polymers (CFRPs) are key materials in the aerospace and automotive industries, and laser micro/nano processing technology has attracted considerable attention in recent years. Machine learning has also demonstrated significant application potential in this precision processing process. Challenges in CFRP processing include delamination, burrs, and tears from traditional mechanical methods [78]. Laser processing offers advantages of non-contact, high precision, and easy automation, but complex interactions with composites can cause HAZ, fiber pull-out, and delamination. Therefore, in the field of CFRP laser processing, a significant challenge lies in the complex interplay between laser parameters, material properties, and the resulting surface morphology and mechanical performance. Integrating machine learning can optimize laser parameters and adjust processing conditions in real time, reducing defects and improving quality. However, traditional modeling approaches can be computationally expensive and may not fully capture the nonlinear and stochastic nature of the laser–material interaction. To address these issues, Zhang et al. summarized the relevant applications of artificial neural networks (ANNs), genetic algorithms (GAs), and Bayesian optimization [79]. ANNs are used to simulate the nonlinear relationship between laser processing parameters (e.g., power, speed) and output quality (e.g., surface roughness, cutting efficiency). These networks are trained on experimental data to predict the optimal settings for specific processing requirements. Genetic algorithms are employed for multi-objective optimization, searching for optimal parameter combinations to balance conflicting objectives, such as maximizing processing speed while minimizing surface defects. Bayesian optimization is employed to efficiently explore the parameter space and identify regions with potential for performance improvement. These data-driven methods significantly enhance the predictability and controllability of CFRP laser processing, reduce trial-and-error iterations, and improve overall manufacturing efficiency, enabling the handling of complex interactions and real-time adaptation to changing conditions. However, challenges remain in terms of data requirements, computational costs, and the interpretability of model outputs.

Machine learning data-driven modeling exhibits powerful nonlinear fitting and predictive capabilities in laser processing. For instance, Zhang et al.’s ANN achieved 98% classification accuracy and R^2^ = 0.99681 [46], while Luo et al.’s DCNN attained R^2^ > 0.96 [80]. By mining implicit patterns from massive process data, these methods bypass complex physical equations, proving ideal for multi-parameter coupled process optimization. However, they heavily rely on training data volume and quality; predictive reliability drops significantly when parameters exceed training ranges. Additionally, their black-box nature limits interpretability, hindering physical mechanism revelation. These limitations drive the exploration of hybrid modeling, integrating physical principles with data-driven approaches.

### 2.2. Physics-Driven Modeling

While data-driven modeling leverages experimental datasets to map process–property relationships, its reliance on massive high-fidelity data poses significant challenges in laser processing due to the scarcity and high cost of empirical measurements. To mitigate data scarcity, physics-based computational methods—such as the finite element method (FEM) for thermo-mechanical simulations [81] and CALPHAD for thermodynamic modeling [48]—generate synthetic datasets by encoding domain knowledge (e.g., heat transfer equations, phase-transition kinetics). However, constructing high-resolution FEM models demands substantial computational resources and time, especially for multi-physics coupling scenarios like laser–material interactions involving fluid dynamics, phase changes, and thermal stresses. Emerging as a paradigm shift, physics-informed neural networks (PINNs) bypass the need for precomputed datasets by directly embedding governing partial differential equations—such as energy conservation laws—into the loss function of neural networks [82]. This approach not only eliminates the cost of data generation but also enhances interpretability by enforcing physical consistency, bridging the gap between black-box data fitting and white-box theoretical models. Together, FEM, CALPHAD, and PINNs form a tiered strategy to address data scarcity while grounding predictions in first principles, ultimately advancing both efficiency and robustness in laser manufacturing optimization.

**Figure 4:**
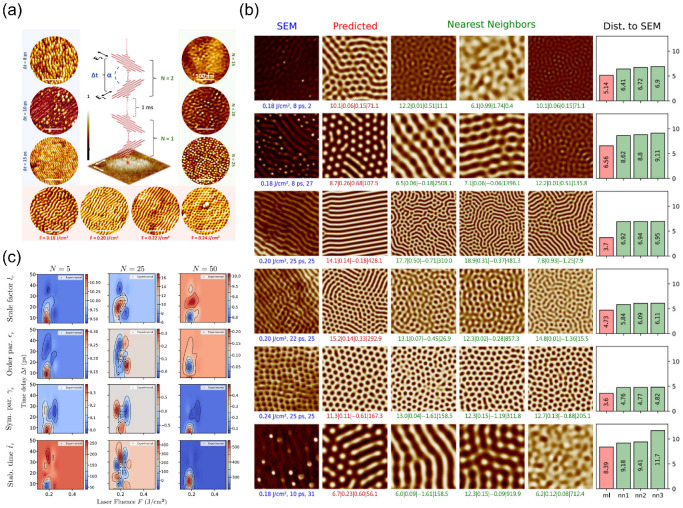
(**a**) Self-organized nanoscale patterning induced by ultrafast laser double pulses, showing the evolution of surface structures under varied inter-pulse delay (Δt), laser fluence (F), and number of pulses (N), as characterized by AFM in 2D and 3D modes. Scale bars: 500 nm; Reprinted with permission from Ref. [49]. Copyright 2025, American Physical Society; (**b**) ML prediction of nanoscale patterns from unseen SEM images. Each row shows an experimental SEM image, the ML-predicted output, and three nearest solver-generated neighbors; Reprinted with permission from Ref. [49]. Copyright 2025, American Physical Society; (**c**) relationship between generalized Swift-Hohenberg equation parameters and laser parameters predicted by ML model. Reprinted with permission from Ref. [49]. Copyright 2025, American Physical Society.

Brandao et al. proposed a hybrid strategy combining physics-driven models and ML to predict laser-induced nanoscale surface patterns [49]. They employed the generalized Swift–Hohenberg equation to describe laser-driven self-organization and trained deep convolutional networks to learn relationships between experimental data and model parameters. Specifically, laser parameters (e.g., fluence, pulse interval, pulse count) served as inputs, while ML models predicted nanoscale pattern morphology and feature dimensions. Figure 4a demonstrates self-organized patterns under varied laser parameters. By adjusting fluence, pulse interval, and pulse count, distinct nanostructures emerged—including nanogratings, nanovoids, and nanocones—providing critical training data for ML models. Figure 4b further validates ML model accuracy in predicting surface nanopatterns. Comparative analysis between experimental and predicted images confirms precise morphology and dimensional predictions. Additionally, the model identifies multi-scale patterns crucial for understanding self-organization physics. Figure 4c reveals relationships between laser parameters and Swift–Hohenberg equation coefficients predicted by ML. These parameters elucidate self-organization mechanisms and guide laser parameter optimization.

Zhang, Z. et al. developed a “physics-guided ML framework” by solving electron rate equations to compute intermediate physical quantities (e.g., plasma peak density, duration), which were incorporated as additional input dimensions for support vector regression (SVR) [50]. This strategy significantly improved predictions for material removal rate and depth in laser-induced plasma micro-machining (LIPMM) (test set R^2^ > 92%, MAE reduced by 78%). Concurrently, it guided genetic algorithm (GA) optimization, boosting processing efficiency by 15.3%. Velli et al. demonstrated a physical-driven modeling approach that leveraged multi-scale simulations to understand complex laser–material interactions [83]. This approach involved integrating modules that accounted for processes such as laser energy absorption, electron excitation, heat transfer, phase transformation, and resolidification. To enhance the predictive power of these physical models, Velli et al. augmented experimental data with simulated data, using an information-theoretic metric to identify regions of high uncertainty. This hybrid approach improved the accuracy of predictions, particularly in critical transition regions where surface structures changed. The study highlighted the benefits of combining physical modeling with machine learning techniques, allowing for a more comprehensive understanding of laser-induced surface modifications while improving the reliability of predictions through data augmentation. Zhang, J. et al. quantified keyhole dynamics via synchrotron X-ray imaging, building Gaussian process regression (GPR) models to predict laser absorptivity [84]. Integrating predictions into thermofluidic CFD models reduced keyhole depth prediction errors from 30% (raytracing) to 10%. Such research demonstrates ML’s role in bridging microscopic physics (e.g., precipitation kinetics) and macroscopic processing performance.

Vo et al. established an automated data acquisition system extracting melt pool features via Otsu thresholding and Sobel edge detection, training Bayesian-regularized neural networks (BR-NNs) to optimize melt pool temperature and processing efficiency (prediction error < 4%) [81]. These works highlight the efficacy of hybrid “physical simulation + experimental calibration” data generation strategies for small-sample challenges. Parodo et al. utilized finite element analysis (FEA) to optimize the laser texturing process for CFRP bonding [52]. FEA was employed to identify high-stress regions within the adhesive joint, enabling targeted laser pretreatment that reduced processing time while preserving joint strength. This approach is well suited for scenarios where the physical behavior of the system can be precisely modeled based on established physical laws and material properties. In contrast to data-driven methods, FEA provides a transparent and interpretable model. However, it necessitates a thorough understanding of the system’s physics and can be computationally demanding for complex geometries or dynamic conditions. The advantages of FEA include high accuracy and reliability in performance prediction, while the disadvantages involve the requirement for substantial computational resources and expertise in physical modeling.

Su et al. proposed a metallurgy-guided ML framework for additive manufacturing, using CALPHAD high-throughput calculations to derive solidification freezing range (SFR) and hot-cracking susceptibility (HCS) [48]. Trained random forest (RF) surrogate models coupled with NSGA-III multi-objective optimization designed novel martensitic stainless steel. After laser-directed energy deposition (LDED), in situ carbide precipitation and martensitic transformation enabled tunable yield strength (1062–1769 MPa) and uniform elongation (2.1–11.7%). Addressing high costs in physical model calibration, Shafaie et al. constructed a data-driven system integrating finite element modeling (FEM), deep neural networks (DNNs), and GA [85]. FEM-generated datasets on elastoplastic and fracture parameters trained DNNs to predict material bending behavior, while GA minimized errors between experiments and simulations. This system achieved accurate fracture prediction for Ti-6Al-4V alloy (RMSE < 10%) with minimal experimental data.

**Figure 5:**
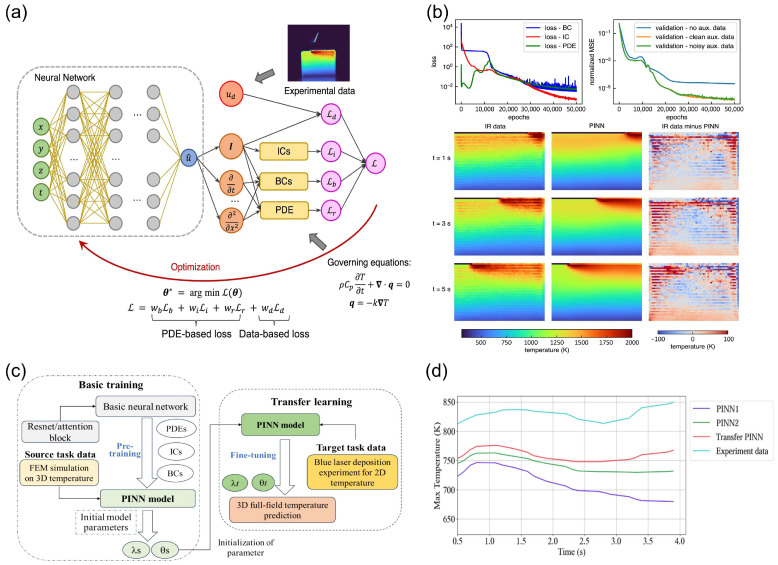
(**a**) Schematic of PINNs modeling for additive manufacturing; Reprinted with permission from Ref. [82]. Copyright 2023, Spring Nature; (**b**) PINN model test and full-field temperature prediction results; Reprinted with permission from Ref. [82]. Copyright 2023, Spring Nature; (**c**) a framework for transfer learning combined with PINNs; Reprinted with permission from Ref. [51]. Copyright 2025, Elsevier; (**d**) temperature prediction comparison between Transfer PINN and other PINNs; Reprinted with permission from Ref. [51]. Copyright 2025, Elsevier.

In the realm of physical-driven modeling, a notable advancement is the integration of physics-informed neural networks (PINNs) in laser processing simulations, as demonstrated in the study by Liao et al. [82]. PINNs, a hybrid approach combining physical laws with data-driven learning, have shown significant potential in addressing the challenges of data scarcity and enhancing model interpretability. Figure 5a illustrates the detailed workflows of the PINN-based modeling framework, which combines the governing equations of heat transfer with partially observed temperature data to predict the full-field temperature history and identify unknown material and process parameters. The PINNs modeling workflows begin with the definition of the governing equations that describe the physical phenomenon, such as the heat transfer equation. These equations are then encoded into the neural network architecture as soft constraints through the loss function, which includes the residuals of the partial differential equations (PDEs), boundary conditions (BCs), and initial conditions (ICs). During training, the network learns to minimize this loss function, effectively approximating the solution to the PDEs. A crucial distinction between PINNs and traditional data-driven models lies in their handling of physical laws. While data-driven models rely exclusively on large amounts of labeled data to learn patterns and make predictions, PINNs incorporate physical laws directly into the learning process. This integration ensures that the model is not only data-efficient but also physically consistent, improving its interpretability and reliability. In scenarios where high-quality experimental data are scarce, PINNs can leverage even limited datasets to make accurate predictions. Liao et al.’s study demonstrates the effectiveness of this approach through various numerical and experimental examples. For instance, the use of auxiliary data, as shown in Figure 5b, significantly accelerates the training process and enhances prediction accuracy. Moreover, Figure 5b presents the results of full-field temperature prediction in an experimental case, where the PINN-based hybrid model successfully inferred the temperature history from partially observed data. This highlights the flexibility of PINNs in real-world applications. Peng et al. proposed a transfer learning-based PINNs framework for predicting the 3D temperature field during blue laser deposition [51]. This framework effectively addresses data scarcity issues and significantly improves prediction accuracy by pre-training on a source task and fine-tuning on a target task. Figure 5c illustrates the overall framework of this method, highlighting the importance of the pre-training and transfer learning stages. Through this strategy, PINNs can quickly converge on limited datasets while maintaining high precision. Furthermore, Figure 5d compares the performance of different PINN models in predicting the maximum temperature curve at the center of the molten pool. The results demonstrate that the Transfer PINN model, which further incorporates transfer learning, exhibits higher accuracy and reliability in temperature prediction. The Transfer PINN model reduces training time and enhances generalization to unseen data by leveraging pre-trained knowledge. A deeper analysis of PINNs as tools to address data scarcity and improve interpretability reveals that PINNs effectively reduce the model’s reliance on large amounts of labeled data by integrating physical laws. Additionally, PINNs’ parallel computing capabilities and reduced iteration requirements address efficiency issues in temperature field calculations. However, PINN training still depends on the accurate specification of initial and boundary conditions, and for complex multi-physics problems, the design of the model architecture and loss function requires further optimization. Similarly, Zhu et al. created physics-informed neural networks (PINNs) using Heaviside functions to enforce hard boundary conditions, satisfying Dirichlet constraints [86] while coupling Navier–Stokes equations with energy conservation laws. With only 216 datasets, PINNs achieved precise predictions of melt pool temperature and hydrodynamics. Such methods bridge process parameters and performance through physical variables, reducing dependency on big data.

Machine learning-assisted modeling revolutionizes laser processing by synergizing data-driven adaptability (e.g., ANN/GAN for nonlinear mapping) with physics-informed interpretability (e.g., PINNs embedding conservation laws), overcoming computational cost and mechanistic opacity. Future advancements demand resolving inherent limitations: enhancing ANN’s explainability, improving surrogate optimizers’ extrapolation capability, and enabling edge-computing real-time control [81]. This will drive autonomous laser manufacturing systems toward precision and sustainability frontiers.

## 3. In Situ Monitoring and Feedback Control

### 3.1. In Situ Monitoring

As high-precision, high-dynamic laser processing advances toward ultrafast pulse regimes and heterogeneous material manufacturing, traditional in situ monitoring faces core bottlenecks [87]. Optical signals suffer plasma plume occlusion, losing melt pool morphological details. Acoustic emission features struggle to quantify internal fracture behaviors due to environmental noise interference. Laser scanning and interferometry methods face core challenges in complex operating conditions due to environmental vibrations, thermal disturbances, and material inhomogeneities that cause measurement signal distortion. Thermal imaging exhibits dynamic response hysteresis, constraining millisecond-scale thermal field evolution control. Machine learning systematically reconstructs performance boundaries across five key monitoring domains through its data-driven adaptive feature extraction and multi-physics coupling advantages. In optical monitoring, it overcomes diffraction limits and occlusion interference, enabling real-time 3D melt pool reconstruction at nanoscale resolution [88]. Acoustic analysis precisely deciphers frequency shift patterns in micro-crack propagation via nonlinear feature correlation models [89]. Laser scanning and interferometry significantly enhance keyhole depth inversion accuracy in complex environments through dynamic compensation algorithms [90]. Thermal imaging establishes dynamic deduction architectures for heat-conduction-phase transition responses, strengthening prediction robustness under extreme conditions [59]. Multi-modal fusion constructs holographic perception closed loops from microstructural evolution to macro-morphology through spatiotemporal alignment and knowledge distillation of cross-domain heterogeneous data, expanding process state analysis from “single-point observation” to “field-effect coupling” [91]. This technological leap not only decouples light–thermal–mechanical–chemical interactions across scales in femtosecond laser processing but also empowers monitoring systems with autonomous decision-making capabilities for dynamic sensing strategy optimization.

#### 3.1.1. Optical Image-Based Inspection

In laser processing, in situ optical image-based inspection is critical for ensuring machining precision and quality. Machine learning significantly enhances this process efficiency and accuracy.

**Figure 6:**
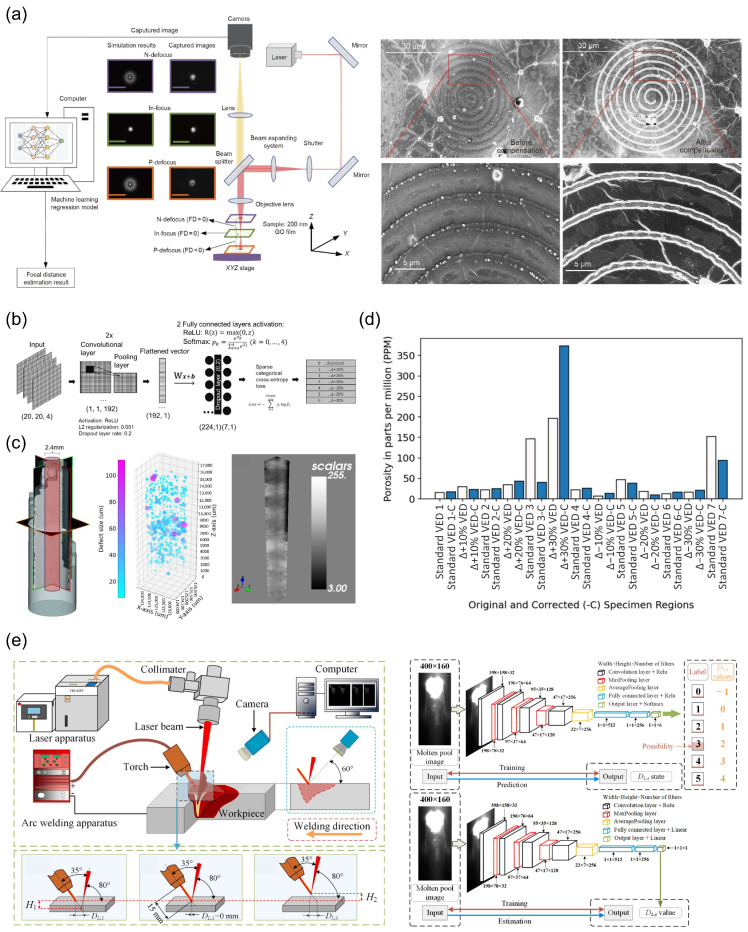
(**a**) Integrated schematic and SEM results of ML-based focus detection in laser nanofabrication. SEM shows structural improvement after compensation. Configuration of laser nanofabrication system; Reprinted with permission from Ref. [17]. Copyright 2025, Elsevier; (**b**) architecture of CNN model [53]; (**c**) defect detection results via XCT system scanning [53]; (**d**) comparison of original and corrected porosity [53]; (**e**) Integrated CNN-based monitoring system for laser-arc distance (D_1_A) showing experimental setup, classification architecture, and regression model. Reprinted with permission from Ref. [93]. Copyright 2024, Elsevier.

Imani et al. proposed a novel deep learning approach for quality control in additive manufacturing (AM) by leveraging layer-wise imaging profiles [92]. The method involves shape-to-image registration, hierarchical dyadic partitioning, and spatial characterization of regions of interest (ROIs) to detect incipient flaws. Experimental results on a drag link joint part demonstrate an accuracy of 92.50 ± 1.03% in flaw detection, with a specificity of 93.85 ± 0.83% and sensitivity of 90.01 ± 1.56%. This approach significantly improves the real-time monitoring and control of AM processes, mitigating interlayer variation and enhancing the overall quality of customized builds. Figure 6a depicts a laser nanofabrication system configuration including laser source, beam splitter, and beam expander, with real-time monitoring via a CMOS camera. Zhang et al. proposed a real-time laser focal spot detection method using ML, achieving 257 nm positioning accuracy through TNN models, surpassing tightly focused laser beam precision [17]. SEM images in Figure 6a demonstrate uniform structure and enhanced contrast after ML-based data compensation. Akmal et al. utilized CNN models (Figure 6b) for defect detection in laser powder bed fusion (PBF-LB), validated by nano-focus X-ray computed tomography (XCT) (Figure 6c) showing defects concentrated in 30% increased VED regions [53]. The model achieved 94% accuracy, with Ti-6Al-4V reducing porosity by 39% at standard VED (Figure 6d). This proves that AI-assisted self-repair enhances manufacturing quality within natural process variations. Cai et al. developed a CNN-based system (Figure 6e) for monitoring laser-arc distance (D_1_A) during hybrid welding [93]. Capturing molten pool images at 30 fps, their classifier achieved 99.83% accuracy while regression models maintained an MAE < 0.3 mm with <2 ms latency. The three methods share fundamental advantages in replacing physical sensors with optical signature interpretation but diverge in technical implementation: spatial regression (TNN) for nanoscale positioning, defect classification (CNN) for process anomalies, and parameter regression (CNN) for thermal flow stabilization, all requiring improved environmental robustness and multi-material validation.

Gobert et al. employed a linear support vector machine (SVM) to classify defects from high-resolution layer-wise images in powder bed fusion, addressing porosity and incomplete fusion by extracting multi-dimensional visual features [54]. This supervised approach achieved >80% accuracy but relied heavily on post-build CT scans for ground-truth labeling, limiting adaptability to dynamic process variations. Xie et al. leveraged a Vision Transformer (ViT) to monitor femtosecond laser machining, using self-attention mechanisms to detect beam translation/rotation and predict thin-film breakthrough [55]. While ViT enabled sub-pixel resolution and multi-parameter tracking, its dependency on computationally intensive affine transformations introduced latency in real-time feedback. Luo et al. employed an image-based DCNN-TL model to analyze in situ photodiode signal data collected during the laser powder bed fusion (LPBF) process [80]. The model was trained on a large-scale image dataset (e.g., ImageNet) and then fine-tuned using data specific to the LPBF process. This approach allowed for accurate predictions of ultimate tensile strength (UTS) and elongation to fracture (EF) for AlSi10Mg and Ti-6Al-4V materials, with prediction accuracies of up to 97.4% for UTS and 95.8% for EF. The implementation of this method involved the generation of layer-wise image datasets from photodiode intensity values, which were synchronized with the laser position. These images were then used as inputs to the DCNN-TL model, which extracted relevant features and made predictions about the mechanical properties of the fabricated parts. The use of multi-sensor data fusion at the feature level further improved the prediction accuracy. This approach is particularly suitable for addressing the challenges of property variability in additively manufactured components due to variations in thermal history and processing parameters. The advantages of this method include its high prediction accuracy, reduced training data requirements, and potential for real-time monitoring and control. However, the complexity of the DCNN architecture and the need for extensive pre-training data are potential limitations.

Tani et al. designed a convolutional autoencoder to process laser speckle patterns, predicting ablation depth and material type via unsupervised feature learning [58]. This method reduced labeling costs but struggled with generalization across heterogeneous materials due to sensitivity to surface roughness variations. Polat et al. utilized a genetically optimized CNN with residual connections and L2 normalization to detect focal deviations on rough surfaces [94]. The nonlinear genetic algorithm (NGA) mitigated overfitting but required extensive hyperparameter tuning. Zheng et al. proposed a semi-supervised convolutional autoencoder (SCAE) for directed energy deposition, constructing domain-level samples to fuse spatiotemporal melt pool features [18]. By activating class spaces with limited labeled data, SCAE reduced annotation costs by 80% while maintaining 83.8% accuracy, though performance depended on precise layer-wise alignment. Schleier et al. applied a Vision Transformer to classify cut interruptions from coaxial thermal images in laser cutting, achieving >96% accuracy in detecting incomplete kerfs [23]. The model’s patch-based self-attention captured melt pool dynamics effectively but faced challenges in generalizing materials with variations in emissivity like aluminum. Qiu et al. developed an NGA-optimized CNN with residual blocks to quantify balling defects in LPBF, using clustering to define seven severity levels [95]. While genetic optimization improved regularization, the method’s reliance on segmented image patches risked loss of spatial context.

These studies collectively demonstrate a paradigm shift from traditional computer vision to deep representation learning yet diverge in supervision strategies and architectural innovation. SVMs and genetic CNNs represent feature-engineered approaches, relying on handcrafted preprocessing to extract structured information, which introduces domain-specific constraints [54,55,95]. In contrast, ViT-based methods and autoencoders exemplify end-to-end representation learning, where attention mechanisms or latent space projections autonomously capture spatiotemporal features, reducing manual intervention but demanding large computational resources. Semi-supervised frameworks bridge the gap between labeled data scarcity and model robustness, while evolutionary algorithms address overfitting through regularization optimization. The critical trade-off emerges between computational efficiency (favoring lightweight CNNs for real-time control) and contextual granularity (requiring ViTs or autoencoders for multi-scale defect analysis). Future advancements must reconcile real-time processing with cross-material generalization. ML has achieved sub-micron spatial resolution and millisecond response in laser processing inspection through TNN, CNN, and Vision Transformers. Future research requires overcoming photon scattering in reflective materials and nonlinear effects in 10 kW + lasers, establishing ML-based predictive control closed loops for zero-defect manufacturing.

#### 3.1.2. Acoustic Emission Monitoring

In the field of laser processing, acoustic signals contain dynamic characteristics of the interaction between lasers and materials (such as melt pool oscillation, crack propagation, and porosity formation). Therefore, in situ acoustic detection is an important non-destructive testing technique that identifies defects in materials by analyzing the acoustic signals generated during processing [19,89,96]. However, traditional acoustic monitoring is susceptible to environmental noise interference and relies on expert experience. Machine learning significantly enhances the real-time performance and accuracy of defect detection by uncovering the relationship between the time–frequency characteristics of acoustic signals and processing conditions. In recent years, three major trends have emerged in this field: single-modal optimization, multi-modal fusion, and innovative decision-making mechanisms.

**Figure 7:**
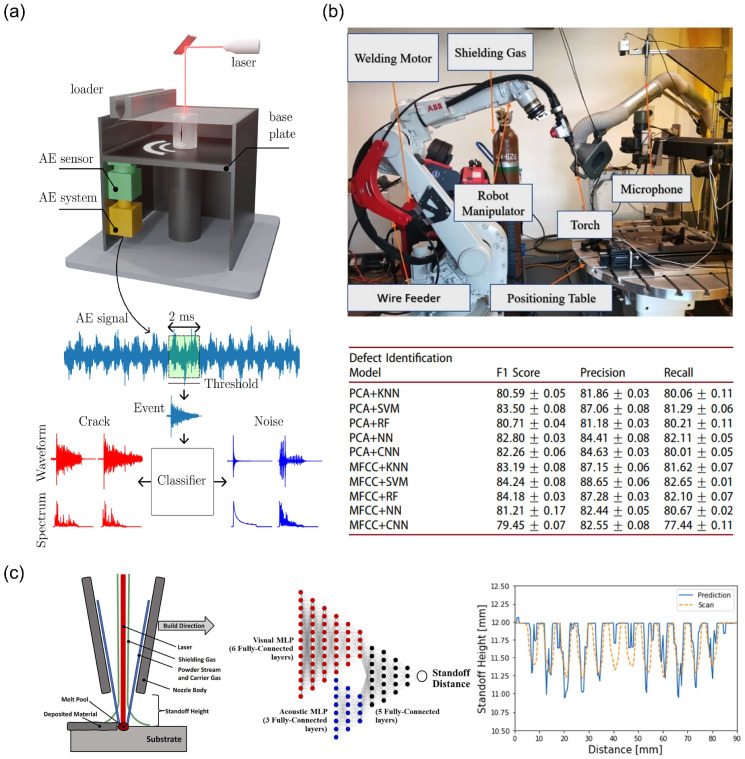
(**a**) Real-time AE signal detection system; Reprinted with permission from Ref. [89]. Copyright 2023, Elsevier; (**b**) comparison of experimental setup and test results for different models [96]; (**c**) integrated framework for real-time standoff estimation in DED: (**left**) schematic of nozzle configuration showing laser/powder focus dependence on standoff distance; (**middle**) multimodal neural network architecture fusing visual and acoustic sensor data; (**right**) validation results demonstrating prediction accuracy against 3D scan measurements. Reprinted with permission from Ref. [19]. Copyright 2025, Elsevier.

Kononenko et al. further optimized the AE signal processing workflow. As shown in Figure 7a, the system monitors AE signals in real time during the LPBF process and filters out AE events exceeding a set threshold [89]. These events are then represented as a finite-dimensional vector of standardized moments and principal components (PCs) and input into a trained machine learning model for classification. Kononenko et al. tested various classification algorithms, including logistic regression, support vector machines (SVMs), random forests, and Gaussian process classifiers. The results showed that in the spectral principal component space, during LPBF experiments, when comparing the performance of logistic regression, SVM, random forests, and Gaussian process classifiers, all models demonstrated high classification accuracy, with the highest reaching 99%.

For nickel-based high-temperature alloy detection, Surovi et al. systematically evaluated acoustic feature extraction methods in Inconel 718 laser processing, compared PCA and MFCC feature extraction effects, and proposed a curvature threshold labeling method to replace subjective visual inspection, optimizing the curvature threshold to divide quality intervals [96]. Figure 7b illustrates the experimental setup and data acquisition process, where acoustic signals are collected via microphones and undergo preprocessing and feature extraction. In terms of machine learning model construction, the study employed five models: KNN, SVM, RF, NN, and CNN. Optimal hyperparameters for each model were determined via five-fold cross-validation, and the performance of 10 combined models was tested on the Inconel 718 dataset. Figure 7b shows the F1 scores, accuracy, and recall rates of different models. The results indicate that the combination of MFCC features and the SVM model achieves the highest F1 score of 84%. The combination of PCA features and the KNN model performs best in the confusion matrix, accurately identifying 186 genuine defective segments.

To overcome the limitations of single modality, multi-sensor fusion has emerged as a new direction. Xie et al. proposed a cross-modal knowledge transfer framework (CMKT) to align acoustic and molten pool visual signals in a shared encoding space [20]. This method achieved 98.6% accuracy in LDED porosity detection and only required acoustic single modality for inference, reducing hardware costs by 50%. Herberger et al. innovatively integrated a coaxial RGB camera with a high-frequency microphone to construct a bimodal MLP prediction architecture [19]. The study utilized the LP-DED system configuration shown in Figure 7c, which includes a coaxial RGB camera and a high-frequency microphone to capture images of the molten pool and collect acoustic signals during processing. Figure 7c illustrates the proposed multi-modal MLP model architecture, which inputs image and acoustic features into their respective MLP networks, performs feature concatenation, and finally predicts the pitch height through a common MLP network. Figure 7c also shows the prediction results in the “wavy pad” experiment, where the multi-modal sensor method accurately captured the changes in the pre-deposited weld bead height. This method demonstrates high-precision prediction capabilities across various geometric shapes and pitch height ranges, with prediction errors within ±200 μm, outperforming traditional methods. Additionally, the multi-modal sensor fusion method exhibits better adaptability and robustness when handling complex geometric shapes and dynamically changing environments.

To address the issue caused by decision delays in machine learning models, Sun et al. developed a time–space fusion decision model (TSFD) that fuses the machine learning output probabilities of the current time step and the previous two time steps through a temporal weighting mechanism, dynamically adjusts the confidence interval based on spatial constraint strategies, and employs three-branch decision theory to divide the decision domain into acceptance, rejection, and delay decision regions, effectively avoiding the limitations of binary misjudgment [97]. In ultra-fast laser drilling experiments, this model significantly reduced the penetration point decision deviation from 68.06 ms to 11.01 ms, successfully suppressing the formation of exit burrs.

The current technical approach demonstrates a continuous evolution from basic feature engineering to intelligent decision-making mechanisms. Early studies such as Kononenko relied on time-domain statistics (mean, variance) and principal component compression [89], while Surovi advanced to frequency-domain MFCC features and curvature threshold label optimization [96]. The work of Herberger marks the maturity of acoustic–visual multi-modal fusion [19]. However, existing challenges are prominently manifested in three aspects: significant decline in model generalization under small sample condition, insufficient real-time performance of multi-modal system, and increased modeling errors in acoustic wave propagation for complex geometric parts. Future research should focus on developing explainable models for acoustic–physical coupling, advancing edge computing acceleration techniques to reduce latency to within 10 ms, and establishing a transfer learning framework across materials and processes to drive this technology toward robust industrial-level applications.

#### 3.1.3. Laser Scanning and Interferometry Inspection

In the field of laser processing, in situ laser scanning and interferometric detection methods have emerged as important quality control tools and have received extensive research and application in recent years.

**Figure 8:**
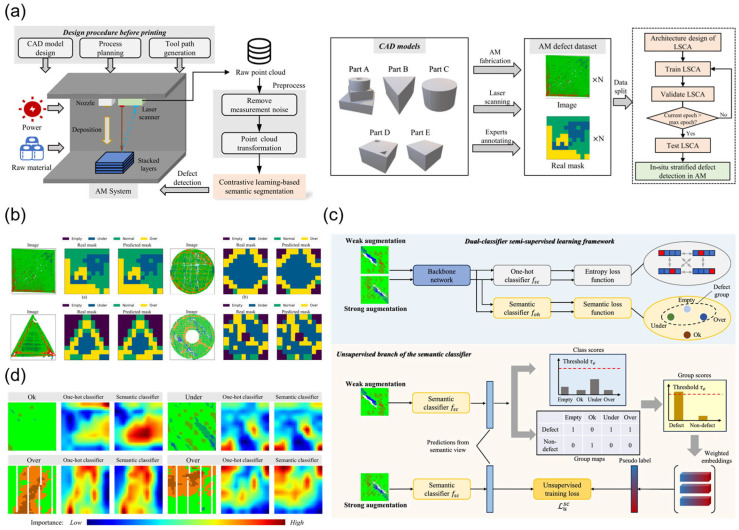
(**a**) AM manufacturing in situ layered defect detection process and defect dataset generation process; Reprinted with permission from Ref. [56]. Copyright 2023, Elsevier; (**b**) LSCA model layered defect detection results; Reprinted with permission from Ref. [56]. Copyright 2023, Elsevier; (**c**) illustrative pipeline of the Dual-Classifier Semi-supervised Learning (DCSL) framework, integrating a one-hot classifier (visual perspective) and a semantic classifier (natural language view) to synergistically leverage both labeled and unlabeled data for enhanced defect detection; Reprinted with permission from Ref. [98]. Copyright 2025, Spring Nature; (**d**) gradient-weighted class activation maps for different defect types. Reprinted with permission from Ref. [98]. Copyright 2025, Spring Nature.

As shown in Figure 8a, in 2023, Kang Wang proposed a contrastive learning-based semantic segmentation model, LSCA, for detecting layered defects in additive manufacturing [56]. LSCA stores and retrieves class-aware semantic memories of images through a defect memory bank and enhances semantic understanding capabilities using class-aware semantic contrast and attention fusion mechanisms. In experiments, LSCA outperformed existing methods in terms of intersection-over-union (IoU) and mean intersection-over-union (mIoU), demonstrating its stable convergence and high detection accuracy when handling imbalanced data. The layered defect detection results of the LSCA model are shown in Figure 8b. This study provides a new solution for defect detection in laser processing, improving detection accuracy and efficiency.

In 2025, Kang Wang et al. proposed an innovative dual-classifier semi-supervised learning method (DCSL), which combines a one-hot encoding classifier and a semantic classifier to train defect class labels from both visual and natural language perspectives, thereby improving defect detection accuracy [98]. The DCSL method utilizes labeled and unlabeled data to improve the quality of pseudo-labels and identifies semantic relationships between class labels through the semantic classifier. Tests on publicly available additive manufacturing defect datasets demonstrate that the DCSL method outperforms current state-of-the-art semi-supervised learning algorithms in defect detection tasks, achieving an accuracy rate of 83.72%. Figure 8c illustrates the DCSL framework, including the supervised branch and unsupervised branch. Figure 8d shows the gradient-weighted class activation maps (Grad-CAMs) for different defect types, indicating that the semantic classifier is more effective in identifying subtle defect changes. LSCA and DCSL collectively drive a paradigm shift in in situ detection technology for laser processing. Future research could explore integrating DCSL’s semantic clustering mechanism into LSCA’s memory update process to achieve “surface-subsurface” defect analysis in high-speed processing scenarios such as laser powder bed fusion (LPBF). By combining traditional non-destructive testing methods like X-ray tomography and optimizing lightweight inference engines, these technologies will ultimately realize the industrial vision of “zero-defect manufacturing”.

Li et al. proposed a fusion method combining a scanning laser source (SLS) with PSO-NN to achieve precise quantification of surface crack depth [99]. This method used a laser beam to scan the crack area and excite surface acoustic waves (SAWs) and established an evaluation model based on the characteristic changes in the interaction between crack depth and SAW. When the laser source crosses the crack, the amplitude and time–frequency characteristics of the reflected wave (rR) and transmitted wave (T) exhibit regular changes. The study extracted time-domain features and time–frequency domain wavelet packet features, verifying the corresponding law between the attenuation of the transmitted wave amplitude and the enhancement of the reflected wave. After selecting sensitive features using a Gaussian process, PSO-NN initial weights were adopted to effectively avoid the local optimum defect of traditional BPNN. Experiments showed that this method achieved an average relative error of <2% in the depth range of 0.3–1.7 mm, and its non-contact characteristics are particularly suitable for high-temperature conditions.

**Figure 9:**
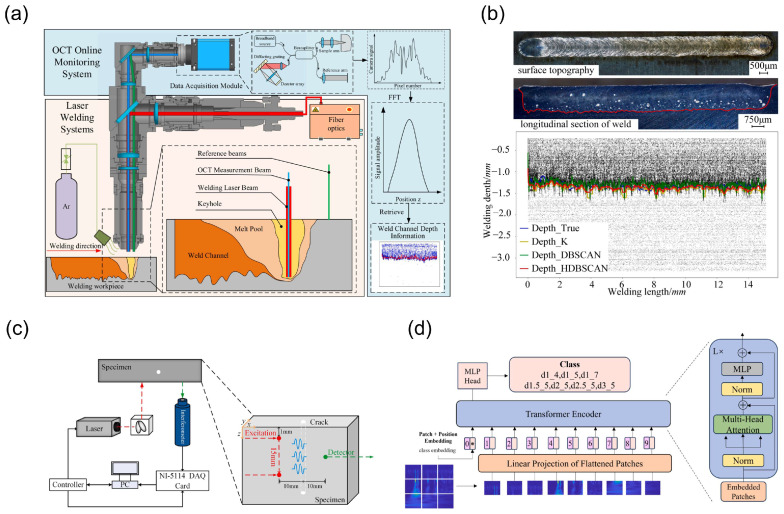
(**a**) OCT laser deep fusion welding inspection system; Reprinted with permission from Ref. [100]. Copyright 2024, Elsevier; (**b**) HDBSCAN algorithm test results; Reprinted with permission from Ref. [100]. Copyright 2024, Elsevier; (**c**) schematic of the laser ultrasonic (LU) experimental system, comprising acquisition equipment and signal acquisition protocol; Reprinted with permission from Ref. [101]. Copyright 2025, Elsevier; (**d**) architecture of the Vision Transformer (ViT) model, illustrating the sequential processing of image patches through linear projection, transformer encoder blocks with multi-head self-attention, and the MLP head for final classification. Reprinted with permission from Ref. [101]. Copyright 2025, Elsevier.

He et al. proposed a laser deep fusion welding depth monitoring method based on optical coherence tomography (OCT), which combines a hierarchical density-based clustering algorithm (HDBSCAN), percentile filtering, and exponential moving average (EMA) to significantly improve the accuracy of depth monitoring [100]. As shown in Figure 9a, this study constructed an integrated OCT laser deep fusion welding detection system. Through the interaction of high-energy laser beams with metallic materials, a melt pool and keyhole are formed, while the OCT system continuously monitors the depth information at the bottom of the keyhole in real time. In the study, the HDBSCAN algorithm was used to perform clustering analysis on the raw OCT data, effectively removing noise. As shown in Figure 9b, the HDBSCAN algorithm eliminates noise while maintaining the accuracy of data points. Compared to the DBSCAN and K-means algorithms, the HDBSCAN algorithm reduces the average error by 46% and 42%, respectively. This method further smooths the data by combining percentile filtering and EMA, improving the accuracy of molten depth curve extraction. Experimental results show that even under continuously varying welding parameters, this method maintains high accuracy, providing robust technical support for real-time monitoring and defect identification during laser welding processes. Wang et al. proposed a method combining variational mode decomposition (VMD) optimized by genetic algorithm weighted permutation entropy (GA-WPE), continuous wavelet transform (CWT), and deep learning fusion models to achieve efficient automatic detection of internal defects in metal equipment [101]. As shown in Figure 9c, the study constructed a laser ultrasonic experimental system and prepared samples with holes of different diameters and depths. The ultrasonic signals were decomposed and reconstructed using the GA-WPE-VMD algorithm, and then the reconstructed signals were converted into time–frequency diagrams using CWT. Subsequently, three deep learning models—ViT, DenseNet121, and ResNet50—were employed for defect detection. As shown in Figure 9d, the ViT model achieved accuracy rates of 99.88%, 97.01%, and 91.7% on the training, validation, and test sets, respectively, with an AUC approaching 100% on the test set. This study demonstrates the effective application of machine learning in laser processing in situ interferometry detection. By combining optimized signal processing methods with deep learning models, the accuracy and reliability of defect detection can be significantly improved. Additionally, this method performs exceptionally well in handling non-stationary and nonlinear signals in complex environments, providing a new solution for non-destructive testing technology in laser processing.

In summary, the core breakthroughs of machine learning-assisted interferometry in situ detection technology lie in the gradual deepening of detection dimensions from the surface to the melt pool and internal layers, feature optimization of PSO-NN [99], noise suppression of HDBSCAN [100], and signal reconstruction of GA-WPE-VMD [101], forming a synergistic evolutionary chain of algorithms. Real-time performance continues to evolve into online diagnostics. Current limitations primarily concern feature stability in high-noise environments, and future research should enhance adaptability in industrial scenarios.

#### 3.1.4. Thermal Imaging Monitoring

In the field of laser processing, in situ thermal imaging is an important process-monitoring technique that evaluates material melting and solidification behaviors by capturing thermal signals during processing [24,59,102,103,104]. In recent years, the introduction of machine learning technology has significantly improved the detection accuracy and reliability of in situ thermal imaging.

**Figure 10:**
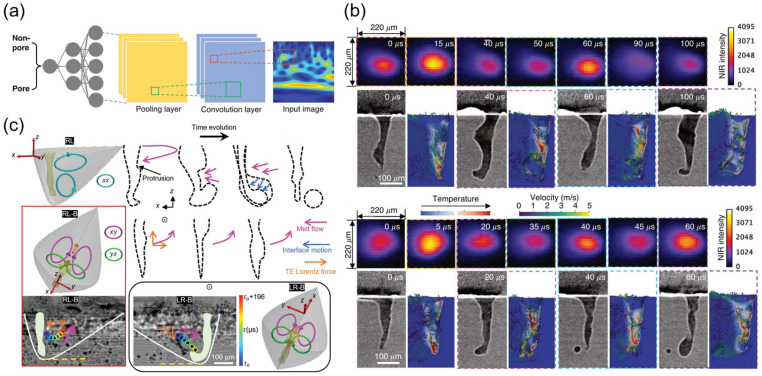
(**a**) CNN model used for thermal imaging detection technology [59]; (**b**) behavioral characteristics of intrinsic oscillations (upper) and disturbance oscillations (lower) in the LPBF process and their impact on keyhole pore generation; arrows indicate thermoelectric (TE) force direction and melt flow, circles highlight flow vortices [59]; (**c**) changes in melt pool flow patterns with and without a magnetic field [104].

Ren et al. developed a machine learning-based in situ thermal imaging method for real-time detection of critical porosity generation during the laser powder bed fusion (LPBF) process [59]. As shown in Figure 10a, Ren et al. utilized a convolutional neural network (CNN) to process thermal imaging data and predict critical pore void formation events. They extracted time-series signals of light emission intensity from the thermal imaging sequence in the critical pore region and generated time–frequency maps via wavelet analysis. These time–frequency maps were segmented and labeled as “voids” or “non-voids” and then input into the CNN for training. The CNN extracted features through a series of alternating convolution and pooling layers, with the final layer classifying the input time–frequency maps. Using this method, they achieved near-perfect detection accuracy for keyhole porosity generation during the LPBF process. Additionally, Ren et al. observed two modes of keyhole oscillations: intrinsic oscillations and perturbed oscillations. As shown in Figure 10b, intrinsic oscillations are caused by changes in the balance between Marangoni forces, surface tension, and backpressure, while perturbed oscillations occur under unstable critical hole conditions, leading to critical hole wall collapse and pore generation. By analyzing these two oscillation modes, they were able to gain a more accurate understanding of the physical mechanisms underlying critical hole pore generation and apply this knowledge to the training of machine learning models.

Fan et al. used high-speed synchrotron X-ray imaging technology to reveal the effects of magnetic fields on keyhole dynamics and melt pool flow during the LPBF process of aluminum alloys [104]. The study showed that applying a transverse magnetic field can significantly reduce keyhole pore area by up to 81%, primarily by altering melt pool flow to suppress the formation of protrusions on the rear wall of the keyhole, thereby stabilizing the keyhole morphology. Figure 10c illustrates the changes in melt pool flow patterns with and without a magnetic field, highlighting the regulatory role of the magnetic field on flow patterns. This provides new insights into the application of machine learning in in situ thermal imaging detection during laser processing, enabling real-time monitoring and adjustment of processing parameters combined with machine learning algorithms to further enhance processing quality and efficiency.

Bostan et al. utilized SHAP-enhanced CNNs to predict subsurface porosity in LPBF Inconel 718 from infrared thermal signatures [103]. Their physics-guided framework incorporates cooling rate, spatter counts, and melt pool area features with a 7 × 7 kernel spatial-context analyzer, achieving 93% balanced accuracy for >34 μm pores. While successfully decoupling lack-of-fusion and keyhole porosity mechanisms, the model relies on destructive validation (serial sectioning) and exhibits high uncertainty near build plate edges due to gas flow effects. The SHAP interpretability represents a significant advancement in model transparency. Yang et al. achieved a 97.25% accuracy rate by combining features from simulated melt pool images and thermal images using a hybrid neural network (HNN) model for defect prediction [24]. The study also proposed a physically supervised network (PSN) model, which improves thermal image features by utilizing simulated melt pool features through feature correlation algorithms, achieving a 96% prediction accuracy rate while maintaining high prediction accuracy and enhancing computational efficiency. Wang et al. proposed combining near-infrared (NIR) thermal imaging and machine learning methods to predict the melt pool cross-sectional morphology during the laser powder bed fusion (LPBF) process [102]. By capturing NIR images of the melt pool during the LPBF process, support vector machine (SVM) and convolutional neural network (CNN) models were used to extract the melt pool’s shape and temperature gradient features, establishing a nonlinear mapping relationship with the solidification laser trajectory cross-sectional geometry. Experimental results showed that the SVM model achieved a prediction accuracy of 76.9%, while the CNN model further improved prediction performance through multi-scale feature fusion, reaching an accuracy of 81.53%. These methods effectively predicted the hidden geometric features of the melt pool, enabling real-time monitoring and quality control of the LPBF process.

In summary, Ren et al.’s research demonstrates the immense potential of machine learning in in situ thermal imaging detection for laser processing [24,59,102,103,104]. By combining high-resolution thermal imaging data with advanced machine learning algorithms, they successfully achieved real-time, high-precision detection of critical pore formation during the LPBF process. This method not only helps improve the manufacturing quality of LPBF technology but also provides new insights and approaches for monitoring laser processing.

#### 3.1.5. Multi-Modal Fusion Monitoring

As high-power laser processing evolves toward ultra-fast and ultra-precise applications, traditional single-mode in situ detection techniques face inherent limitations imposed by their underlying physical principles [60]. Optical imaging is susceptible to interference from plasma shielding, acoustic signals struggle to quantitatively correlate material deformation [105], and thermal imaging cannot capture non-thermal reaction processes. These limitations make it challenging for a single sensing modality to provide comprehensive feedback on processing conditions when analyzing multi-physics coupling effects such as nonlinear absorption and plasma plume expansion induced by femtosecond lasers. Multi-modal fusion technology overcomes this one-dimensional perception bottleneck by aligning heterogeneous sensors in space and time and leveraging their complementary characteristics to establish a holistic perception network linking “physical response-morphological evolution”.

**Figure 11:**
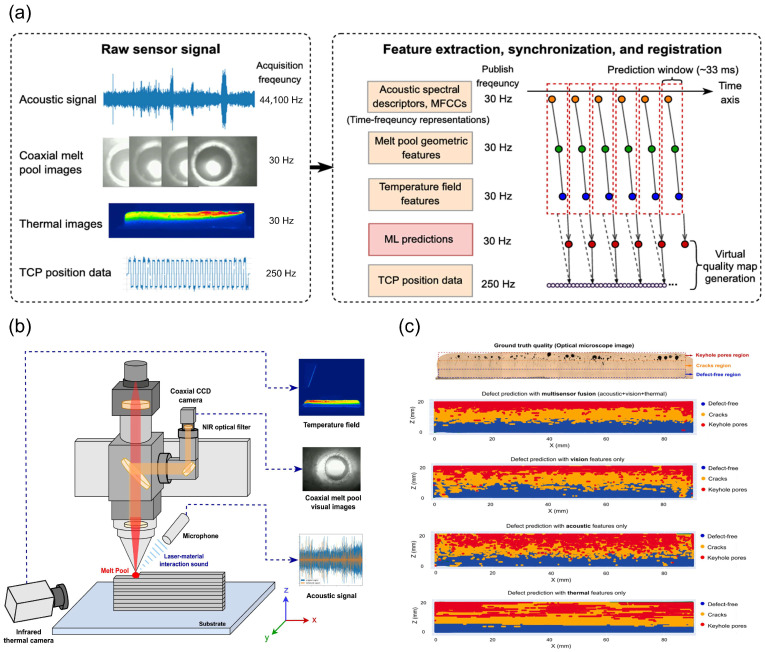
(**a**) Schematic of spatiotemporal synchronization and registration for multisensor data fusion, where colored arrows indicate distinct data flows; Reprinted with permission from Ref. [60]. Copyright 2023, Elsevier; (**b**) multi-modal detection experimental setup; Reprinted with permission from Ref. [60]. Copyright 2023, Elsevier; (**c**) predicted quality within the three-dimensional volume of the printed part. Reprinted with permission from Ref. [60]. Copyright 2023, Elsevier.

Chen et al. proposed a multi-sensor fusion digital twin (MFDT) framework for local quality prediction in robotic laser-directed energy deposition (L-DED) processes [60]. This framework achieves high-precision defect prediction by synchronizing and registering multi-sensor features from coaxial melt pool visual cameras, microphone sensors, and off-axis short-wave infrared thermal imagers. As shown in Figure 11b, the experimental setup integrates three types of sensors. The thermal infrared camera monitors the temperature field of the printed component, the coaxial CCD camera monitors the melt pool geometry, and the microphone sensor captures the sound of laser–material interaction. The data from these sensors are spatiotemporally synchronized and registered using real-time robot tool center point (TCP) position data, as shown in Figure 11a. This spatiotemporal data fusion method precisely captures the complex melt pool behavior during the processing, enabling early detection and prediction of defects. Finally, by training a supervised machine learning model, the research team generated a virtual quality map, as shown in Figure 11c, which displays predicted quality values within the three-dimensional volume of the printed part. The virtual quality map highly matches the quality observed in actual optical microscope (OM) images, validating the high accuracy and reliability of the multi-sensor fusion method in defect prediction.

In summary, Chen et al.’s research significantly improved the accuracy and efficiency of defect detection in laser processing through multi-sensor fusion and spatiotemporal data synchronization technology [60], providing new solutions for quality control and optimization in the field of laser processing.

### 3.2. Feedback Control

Real-time monitoring and closed-loop control of laser processing are key technical bottlenecks in improving manufacturing precision. In recent years, machine learning has made breakthroughs in quality control systems by analyzing multi-modal data such as melt pool dynamics and temperature field distribution [23,60]. By integrating modeling, in situ detection, and feedback control, laser processing systems have achieved a closed-loop “perception-analysis-execution” capability, enabling autonomous decision making for critical processes such as melt pool dynamic control and microcrack detection. Specifically, the feedback control process in laser processing typically includes several key steps: First, establish a nonlinear mapping relationship between processing parameters and outcomes through physics-driven and data-driven modeling methods, predicting melt pool behavior and potential defects during the process [75,82]. Second, use in situ detection techniques, such as optical imaging, acoustic emission monitoring, laser scanning, and interferometry, to capture physical signals in real time during processing and perform feature extraction and defect identification using machine learning algorithms [17,56,96]. Finally, feed the in situ detection data back into the control system, dynamically adjusting laser parameters such as power and scanning speed through adaptive control, to optimize the processing and reduce defect formation, thereby improving processing quality and efficiency [22]. This integrated feedback control strategy not only enhances the accuracy and reliability of laser processing but also opens up new possibilities for the intelligent development of laser processing technology.

Miyaji et al. developed a real-time monitoring and active feedback control system for the fabrication of high-quality nanostructures on glass surfaces using femtosecond laser pulses [63]. The system utilizes changes in the surface reflectance and transmittance during laser irradiation to dynamically adjust the laser power. This is achieved through a proportional–integral–derivative (PID) control system, which maintains the desired setpoints for reflectance and transmittance based on the measured values. The closed-loop control strategy ensures uniformity and high quality of the nanostructures, significantly reducing the defect ratio. The advantages of this method lie in its high precision and automation capabilities, enabling real-time adjustment of laser parameters to adapt to changes in processing conditions, thereby guaranteeing the uniformity and high quality of nanostructures. However, the system also has some drawbacks, such as the complexity of the optical setup and the high sensitivity to surface morphology, which may affect the stability and reliability of the system. Overall, this method provides a powerful tool for intelligent laser processing but still requires further optimization to meet more complex fabrication requirements.

Kwon et al. developed a deep neural network (DNN) for melt pool image classification in metal additive manufacturing [21]. The model takes 60 × 60-pixel melt pool images as input and employs a 10-layer hidden layer structure with the number of nodes decreasing across layers. Within the 100–350 W laser power range, its classification error rate for 13,200 test images was below 1.1%, significantly outperforming the traditional pixel intensity summation method (which had an error rate of 5–20% at power levels above 200 W). The breakthrough of this model lies in its ability to precisely capture the blurred edges and complex morphological features of the molten pool (such as the extent of the heat-affected zone and spatter distribution). By monitoring the deviation between predicted and actual power in real time (with a threshold set at 3%), it can locate regions with abnormal microstructures, opening up new avenues for non-destructive defect screening. In the field of nanoscale processing, Mohanavel et al. proposed the AlexNet-guided femtosecond laser processing framework (AlexNet-GFLP), which achieves dynamic parameter optimization [57]. This framework inputs laser–material interaction images into an improved AlexNet model (with the output layer adjusted for regression tasks) to predict the optimal pulse energy and scanning speed in real time. Experimental validation shows that material removal rate is improved by 20% and feature size accuracy by 15% compared to traditional methods, attributed to the model’s optimization of energy absorption efficiency. Notably, by real-time parameter adjustment to suppress thermal damage, this technology simultaneously reduces residual stress in the material, ensuring the mechanical stability of nanostructures.

**Figure 12:**
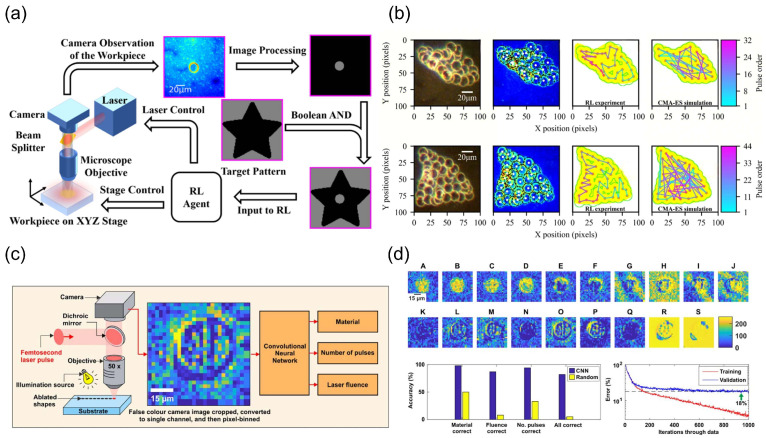
(**a**) Reinforcement learning application process. Arrows indicate the direction of data flow for imaging and control [22]; (**b**) comparison of real-world laser machining results and algorithm-generated toolpaths: camera images (**left**) show actual machined patterns, while visualizations (**right**) represent the initial target shape in yellow [22]; (**c**) CNN-based feedback control experiment diagram, where arrows indicate the direction of data flow from laser processing to camera imaging and subsequent CNN analysis; Reprinted with permission from Ref. [106]. Copyright 2018, IOP Publishing Ltd.; (**d**) validation results of the trained CNN: letters A–S denote the 19 ground-truth parameter categories, and the blue-yellow color gradient represents pixel intensity in the processed single-channel sample images (**upper**). CNN recognition accuracy and fitting error (**lower**). Reprinted with permission from Ref. [106]. Copyright 2018, IOP Publishing Ltd.

Xie et al. proposed a method using reinforcement learning (RL) to control the laser processing process [22]. This method achieves automatic tool path design and high-precision processing results by real-time detection and compensation of error actions. As shown in Figure 12a, the system uses a laser system to process the target material and captures the workpiece image in real time using a CMOS camera. The RL agent is trained in a virtual environment to learn how to select the optimal laser pulse position to maximize processing effectiveness. In physical experiments, the RL agent observes workpiece images and adjusts the laser pulse position in real time to compensate for machine errors, such as platform vibrations. Figure 12b demonstrates the RL agent’s performance in real-world experiments. The RL agent can automatically generate tool paths based on randomly generated target patterns and self-correct during processing. This method not only improves the accuracy and efficiency of laser processing but also reduces the need for human intervention. With the assistance of machine learning, the laser processing process has become more intelligent and automated, bringing new possibilities to the field of high-precision manufacturing. Mills et al. trained a CNN to directly identify material type, laser energy density, and pulse count from a single image of a laser-processed sample without understanding the underlying physical processes [106]. As shown in Figure 12c, the experimental setup involved laser processing of silicon and nickel samples using a titanium sapphire chirped pulse amplification system, while capturing sample images during processing using a CMOS camera. Data collection covered 1800 image pairs across 19 different experimental parameter categories, with 90% used for training the CNN and 10% for validation. Figure 12d shows the CNN’s performance on the validation dataset, with the upper half displaying randomly selected images correctly predicted by the CNN. Despite the subtle differences between adjacent categories, the CNN accurately classified them. Additionally, Figure 12d shows the CNN’s accuracy rates for material, laser energy density, and pulse count recognition, which are 98%, 87%, and 94%, respectively, significantly outperforming the random number generator. Figure 12d also displays the CNN’s fitting error on the training and validation datasets, with the validation error reaching a minimum of 18%, indicating that the CNN did not exhibit severe overfitting during training. Through these experimental results, Mills et al. demonstrated the effectiveness of CNN in femtosecond laser processing monitoring and noted that this method can be extended to other types of laser processing, applicable to any samples observable during the manufacturing process and capable of distinguishing based on the different appearances of experimental parameters. This study provides a new solution for real-time feedback control in laser processing, with the potential to enhance processing accuracy and efficiency.

In summary, machine learning fundamentally enhances laser processing feedback control by enabling adaptive parameter optimization (e.g., PID-controlled power adjustment via reflectance monitoring) [63], real-time defect detection (DNN-based melt pool classification) [21], and dynamic compensation (RL-driven path correction for vibration) [22]. The convergence of machine learning with adaptive control frameworks has transformed laser processing into a self-optimizing discipline, where predictive accuracy and compensatory agility collectively drive toward defect-free fabrication. This evolution underscores the transition from human-supervised adjustments to autonomous cyber–physical systems, charting a clear trajectory for next-generation intelligent laser manufacturing.

## 4. Conclusions

The integration of machine learning with laser micro/nano processing has evolved from single-point process optimization to system-level, end-to-end collaborative integration. Traditional laser micro/nano processing faces numerous challenges, such as nonlinear energy absorption, random plasma effects, and the accumulation of defects during multi-step processes. To address these challenges, various machine learning techniques, including supervised learning, unsupervised learning, semi-supervised learning, and reinforcement learning, have been increasingly applied to the field of laser micro/nano processing. In the modeling phase, machine learning establishes nonlinear mapping between process parameters and processing results. Optimization algorithm-based methods (such as GPR + BO, ANN/GA/BO) are preferred for parameter optimization due to their ability to reduce trial-and-error costs and handle nonlinear problems, particularly for geometric precision control in multiphoton 3D printing; however, these methods generally suffer from a fundamental flaw: the absence of physical mechanisms. For example, ignoring thermal accumulation effects makes it difficult to translate laboratory results into actual production lines. Physically integrated methods (such as PINNs and FEA optimization frameworks) incorporate physical constraints like heat conduction equations, demonstrating advantages in multi-physics coupling predictions. A typical example is blue laser deposition temperature field simulation. However, their computational complexity conflicts sharply with industrial real-time requirements—simplifying equations sacrifices accuracy (e.g., relying on electron velocity equations leads to cross-process failures), while complete modeling is constrained by computational power bottlenecks. Generative models (such as Gen-JEMA) can generate multi-modal melt pool data to assist training, but they remain theoretical explorations due to the insufficient performance of VAE and excessive computational burden. In the in situ monitoring and feedback control phase, by integrating multi-modal sensing and reinforcement learning, the processing system has gained the ability to form a closed-loop “perception-analysis-execution” capability for the first time, enabling key processes such as melt pool dynamic control and microcrack detection to transition from passive response to autonomous decision making. Image recognition methods each have their strengths: CNNs excel in efficient feature extraction and are the mainstay of defect detection (e.g., real-time analysis of LBPF fusion states), but their performance fluctuations in heterogeneous materials expose data dependency issues. ViT has achieved breakthroughs in sub-pixel-level beam movement detection. Small-sample methods (e.g., SVM) have practical value in scenarios such as pore classification during the early stages of material development. Dimension reduction methods (such as semi-supervised CAE) have emerged in low-contrast melt pool monitoring through spatiotemporal feature fusion. RL demonstrates autonomous decision-making potential in path dynamic correction.

In the face of these challenges, active exploration is underway. To address the bottleneck of computational efficiency, researchers are working to develop more lightweight physical fusion models, such as refining the core parts of key physical equations into simplified modules that can be embedded in neural networks and combining them with dedicated hardware acceleration to meet the millisecond-level response requirements of production lines while maintaining physical meaning. For data dependency and generalization issues, a promising direction is to transform physical rules themselves into data generation engines, using known laser–material interaction principles to synthesize large amounts of realistic training data. This not only alleviates the pressure of insufficient real data but also enables the model to adapt more effectively when faced with new materials or processes. To bridge the gap between simulation and reality, it is necessary to closely integrate reinforcement learning training environments with real-world physical sensor feedback, creating a “virtual-reality” hybrid training environment. This allows the intelligent agent to learn foundational strategies in simulation and then fine-tune and validate them through minimal real-time feedback during actual processing, gradually enhancing its decision-making robustness in real-world complex environments.

Machine learning empowers laser micro/nano processing by redefining the basic logic of laser processing. When algorithms transform the complex interactions between light and matter into programmable physical laws, humanity achieves a paradigm shift from “experience-based” to “cognitive autonomy” in manufacturing for the first time. This process not only redefines the precision boundaries of advanced micro/nano manufacturing but also catalyzes the continuous expansion of industrial civilization into deep space exploration and a sustainable future. Machine learning is no longer an isolated technical tool; it is becoming the intrinsic “intelligent core” of laser micro/nano processing systems. This integration will drive us from the traditional model of relying on trial and error based on experience toward a new era of precision manufacturing based on data and intelligence. It not only holds the potential to address the current yield and efficiency bottlenecks in high-end manufacturing but will also unlock unprecedented processing capabilities, enabling the production of more complex and superior-performing micro/nano devices. These devices will provide core support for next-generation information technology, biomedicine, and advanced energy sectors. This is not merely a technological advancement but a revolutionary shift in manufacturing philosophy.

## Figures and Tables

**Table 1:** The ML methods commonly used in laser micro/nano processing for each flow and their characteristics.

Processing Flow	ML Methods	Advantages and Disadvantages	Purpose	Types of Laser Processes	Reference
Modeling	GPR + BO	Adv: Suitable for low-dimensional nonlinear problems; Disadv: Ignores thermal accumulation effects	Optimize 2D geometric accuracy	Projection multiphoton 3D printing	Johnson et al. [45]
	ANN/GA/BO	Adv: Reduces trial and error; Disadv: High data/computation cost, poor interpretability	Optimize params for surface/mechanical quality	CFRP laser processing	Zhang et al. [46]
	Gen-JEMA (AE/VAE + BO)	Adv: Suitable for multi-modal data prediction; Disadv: VAE underperforms, computational complexity	Melt pool geometry prediction and data generation	LDED	Ferreira et al. [47]
	RF + CALPHAD calculations	Adv: Handles high-dimensional data and large number of features; Disadv: Long training and prediction time	Design martensitic steel with tunable strength/ductility		Su et al. [48]
	Deep Convolutional Networks	Adv: Suitable for image prediction; Disadv: Requires extensive training data	Predict nanoscale pattern morphology and feature dimensions	Laser-induced nanoscale patterning	Brandao et al. [49]
	SVR + Physics-guided inputs	Adv: Suitable for complex process optimization of small samples with well-defined physical mechanisms; Disadv: Dependency on electron rate equations for intermediate physics	Predict material removal rate and depth	LIPMM	Zhang et al. [50]
	Transfer Learning-based PINNs	Adv: Reduces training time via pre-training; Disadv: Adaptive bottlenecks in physical mechanism migration	Predict 3D temperature field	Blue laser deposition	Peng et al. [51]
	FEA-guided optimization	Adv: Suitable for multi-physics field coupling parameter optimization problems; Disadv: Computationally demanding for complex geometries	Identify high-stress regions for targeted pretreatment	CFRP laser texturing	Parodo et al. [52]
In situ Monitoring	TNN	Adv: Suitable for multi-dimensional feature extraction; Disadv: Requires high-resolution CMOS imaging	Real-time focal spot positioning	Laser nanofabrication	Zhang et al. [17]
	CNN	Adv: Suitable for high-precision real-time analysis of images; Disadv: Performance drops in heterogeneous materials	Defect detection	LBPF	Akmal et al. [53]
	SVM	Adv: Suitable for classification problems with small sample data; Disadv: Dependent on processing parameters	Classify porosity/incomplete fusion		Gobert et al. [54]
	ViT	Adv: Sub-pixel resolution; Disadv: Computationally intensive affine transformations cause latency	Detect beam translation/rotation and predict thin-film breakthrough	Femtosecond laser machining	Xie et al. [55]
	Semi-supervised CAE	Adv: Low training costs; Disadv: Computational burden of image alignment and spatiotemporal alignment	Fuse spatiotemporal melt pool features	LDED	Zheng et al. [18]
	LSCA	Adv: Suitable for high-noise, low-contrast, multi-scale scenes imbalance; Disadv: Computational efficiency and real-time bottlenecks	Semantic segmentation of layer defects	Additive manufacturing layer defects	Wang et al. [56]
Feedback Control	RL	Adv: Autonomous error correction, reduces human intervention; Disadv: Training requires virtual environment simulation	Real-time tool path correction and vibration compensation	High-precision laser machining	Xie et al. [22]
	AlexNet-Guided Framework	Adv: Suitable for parametric co-optimization; Disadv: Real-time bottlenecks with high-resolution inputs	Dynamic optimization of pulse energy and scan speed	Nanoscale laser processing	Mohanavel et al. [57]
